# Energy-Screened Many-Body Expansion for Protein–Ligand Interactions: Examining Convergence for Metalloenzymes Through Seven–Body Interactions

**DOI:** 10.1021/acs.jctc.6c00190

**Published:** 2026-03-30

**Authors:** Paige E. Bowling, Dustin R. Broderick, John M. Herbert

**Affiliations:** † Department of Chemistry, University of Michigan, Ann Arbor, Michigan 48109, United States; ‡ Biophysics Graduate Program, 2647 The Ohio State University, Columbus, Ohio 43210, United States; § Department of Chemistry & Biochemistry, 2647 The Ohio State University, Columbus, Ohio 43210, United States; ∥ Department of Chemistry, University of Chicago, Chicago, Illinois 60637, United States

## Abstract

Fragment-based quantum chemistry is a powerful strategy for calculating protein–ligand interaction energies using quantum chemistry methods. Rigorous convergence often requires hundreds of atoms in the protein binding-site model, especially if that model is constructed using distance-based criteria to select amino acid residues, while three- and four-body calculations exhibit instability related to combinatorial proliferation in the number of subsystem calculations. Here, we report an energy-based screening protocol for the many-body expansion applied to protein–ligand interactions, implemented in the open-source Fragme∩t code. Using a combination of aggressive screening based on semiempirical quantum chemistry, with an improved graph-theoretical algorithm to eliminate unimportant subsystems, we are able to perform *n*-body calculations up to *n* = 7 using density functional theory in triple-ζ basis sets. Distance cutoffs further reduce the cost without compromising accuracy. Rapid and stable convergence of the many-body expansion is obtained by *n* = 4, for a pair of metalloenzymes in which a divalent ion coordinates directly to the ligand. As compared to previous results that relied solely on distance cutoffs, oscillations in the *n*-body corrections are reduced or eliminated, although residual errors remain in one case. This work demonstrates that benchmark-quality protein–ligand interaction energies can be systematically converged using a method with excellent parallel efficiency and scalability.

## Graphical abstract

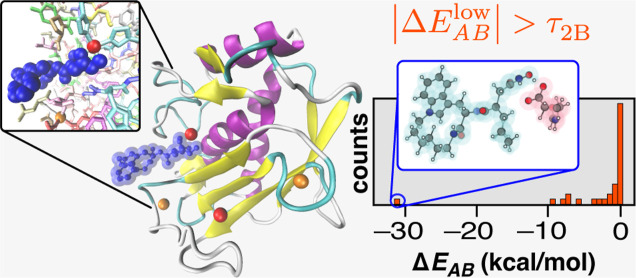

## 1 Introduction

Enzyme inhibitors comprise up to 50% of existing drugs,^1–3^ with noncovalent inhibition as the mechanism pursued by most drug-discovery teams.^4^ Therefore, understanding the molecular physics of protein–ligand (P:L) binding and how it changes upon ligand redesign is a fundamental aspect of computer-aided drug discovery. Quantum chemistry (QC) has the potential to offer insight along these lines,^5^ although P:L interaction energies are slow to converge with respect to the size of the binding-site model, often requiring 400 atoms or more.^6,7^ The same problem affects active-site models of enzymatic reaction energies,^8–10^ and results in large part from inter-residue and protein-to-solvent charge transfer.^9–13^ These effects require a quantum-mechanical description but QC calculations at this scale are challenging, even with density functional theory (DFT), especially given that double-ζ basis sets do not afford converged results.^7,14^ QC calculations at levels of theory beyond DFT are simply intractable without additional approximations.

Fragment-based QC offers a means to this end, and in the present work we examine the many-body expansion (MBE) as a simple paradigm.^15^ It starts from one-body fragment energies ${\left\{E_{A}\right\}}_{A=1,...,N}$ that are systematically corrected for two-body interactions (Δ*E*_*AB*_), three-body interactions (Δ*E*_*ABC*_), etc.:

$$E=\overset{N}{\underset{A=1}{\sum }}\left[E_{A}+\underset{B>A}{\sum }\left(\Delta E_{AB}+\underset{C>B}{\sum }\Delta E_{ABC}+\cdot \cdot \cdot \right)\right].$$ 1

Explicitly, the two- and three-body corrections are

2 $$\Delta E_{AB}=E_{AB}-E_{A}-E_{B}$$

and

$$\begin{array}{ll} \Delta E_{ABC} & =E_{ABC}-\Delta E_{AB}-\Delta E_{AC}-\Delta E_{BC}-\\ & E_{A}-E_{B}-E_{C}. \end{array}$$ 3

Higher-order terms are explicated elsewhere.^16^ If eq 1 is truncated at *n*-body terms, we refer to the resulting approximation as MBE­(*n*).

Whereas MBE­(*n*) calculations on water clusters and ion–water clusters unambiguously demonstrate the importance of three- and four-body corrections,^15–20^ results for P:L interaction energies are more equivocal. Using single-residue fragments for the protein, we have identified cases where three- and four-body corrections were significant and oscillatory, in the sense that the MBE(4) result deviated from a supramolecular benchmark by a larger amount as compared to the MBE(3) approximation. The problematic cases were a pair of metalloenzymes in which a Zn^2+^ cation binds to the ligand. Divalent ions are know to exacerbate model-size effects,^21^ so these are the test systems used in the present work.

Further investigation of these effects has been precluded by combinatorial proliferation of *n*-body subsystems in high-order MBE­(*n*) calculations where the number of unique subsystems grows as $O\left(N^{n}\right)$ . The cost can be mitigated somewhat using distance-based screening, but not to a degree that makes MBE(4) routinely feasible. Thus, MBE­(*n*) calculations face a dilemma: either accept that fragmentation errors cannot be systematically reduced using higher-order terms, or face prohibitive cost (and potential loss of precision) by including terms with *n* ≥ 4.^15–18^

For water and ion–water clusters, we have solved this problem with an energy-based screening procedure.^22,23^ The idea is to cull the *n*-body subsystems using an inexpensive level of theory such as a classical force field or semiempirical QC method.^22–24^ Only those *n*-body corrections that register above a user-defined threshold are evaluated at the target level of theory. For a target accuracy of ∼1 kcal/mol, we find this to be more efficient than distance-based screening,^22^ in part because it incorporates cooperative effects that are omitted by aggressive distance-based thresholding.^24^

For screening based on semiempirical QC, this energy-based approach must be combined with graph-theoretical techniques to eliminate higher-order subsystems, else the cost to evaluate four-body interactions becomes prohibitive even for the low-level screening method. The resulting “bottom-up” screening algorithm^23^ has enabled us to performed converged four-body expansions in (H_2_O)_64_ with *N* = 64 fragments. Of the 680,120 distinct subsystems in a complete MBE(4) calculation with *N* = 64, fewer than 1% were computed at the target level of theory. In this way, expansions with *n* > 4 were rendered feasible,^23^ which exposed some artifacts in DFT calculations related to the interplay of delocalization error and the *n*-body expansion.^19,20^

In the present work, we report an implementation of energy-screened MBE­(*n*) for P:L interactions. We then revisit the metalloproteases 1ZP5 ^25^ and 1MMQ,^26^ in which an inhibitor is bound to Zn^2+^ (Figure 1). These systems were considered in previous work,^6,7^ where we found—unsurprisingly—that the divalent ion creates significant polarization interactions. These cannot be mitigated simply by neutralizing the fragments, as is sometimes done when fragment-based QC is applied to enzymatic systems,^27–29^ because the ligand must be removed in order to compute the intermolecular interaction energy.

**Figure 1:**
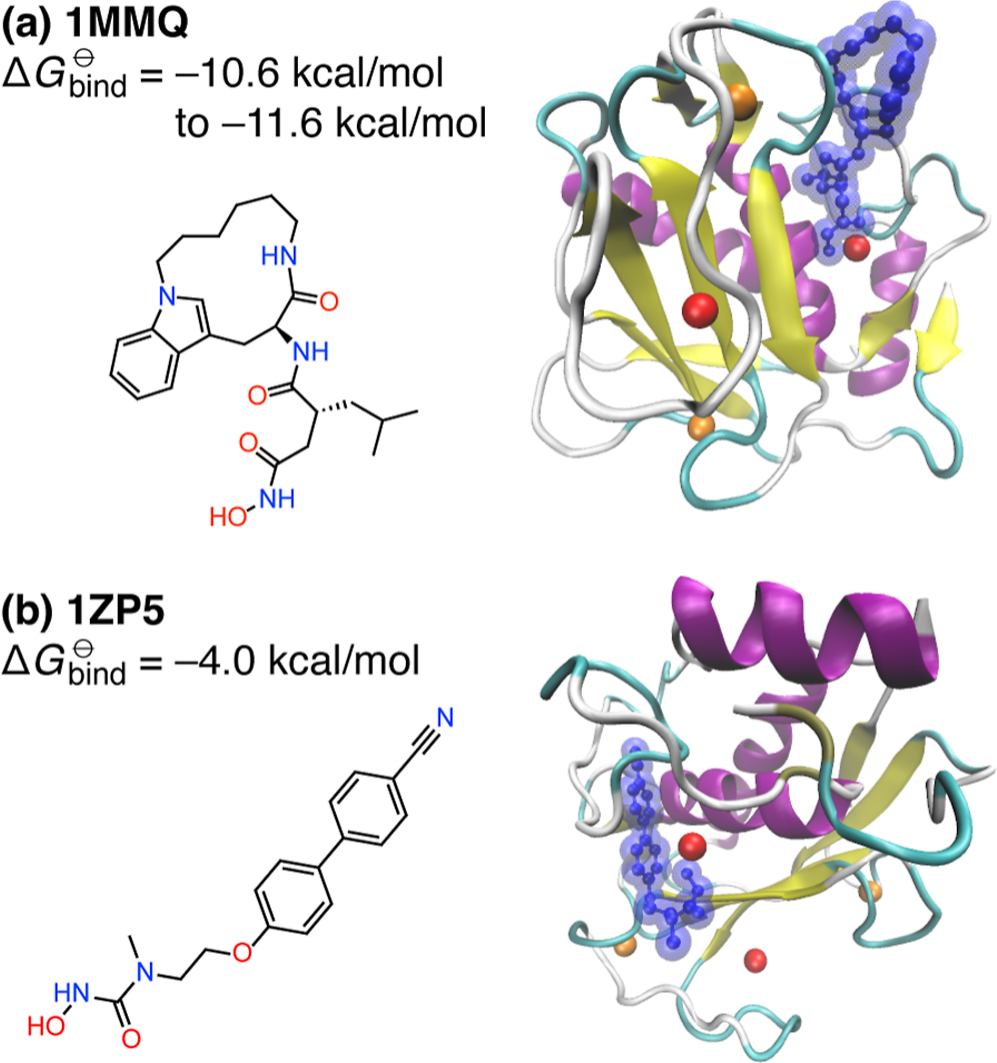
P:L systems considered in this work: (a) matrilysin complexed with a hydroxamate inhibitor (PDB: 1MMQ), and (b) matrix metalloproteinase-8 complexed with a *N*-hydroxyurea inhibitor (PDB: 1ZP5). Chemical structures for the ligands are shown along with crystal structures of the P:L complexes, In the latter, ball-and-stick space-filling ligand models are shown in blue (sans hydrogen atoms), while Zn^2+^ and Ca^2+^ ions appear as red and orange spheres, respectively.

Here, we demonstrate that converged interaction energies can be obtained using low-order *n*-body calculations based on single-residue fragments, if an appropriate screening strategy is employed. To verify convergence, we extend these calculations all the way to *n* = 7, using DFT in triple-ζ basis sets. This illustrates that the previously observed oscillatory behavior arises, in large part, due to accumulation of roundoff error associated with a rapidly growing number of subsystems. More generally, the protocols developed here provide a framework to extend high-accuracy QC to large metalloenzymes, with controllable convergence. While others have started to use fragment-based QC to obtain P:L interaction energies at levels of theory beyond DFT,^30^ convergence with respect to the size of the enzyme model and the treatment of fragmentation have not been demonstrated. The present work provides a paradigm for doing so.

## 2 Theory and Methods

The methodology described here is implemented in Fragme∩t,^31^ an open-source Python application for fragment-based QC that handles fragmentation, subsystem creation, database management, and parallelization for MBE­(*n*) calculations. Fragme∩t interfaces with numerous QC software packages to perform the electronic structure calculations and these interfaces are easy to add or modify.^31^ QC calculations reported in this work were performed using Q-Chem.^32^

Fragme∩t was developed around the idea of screening, which is described in Section 2.2 following a discussion of MBE­(*n*) for the P:L interaction problem in Section 2.1. Setup of the biomolecular models is described in Section 2.3.

### 2.1 Interaction Energies

P:L interaction energies are computed using the supramolecular approach

4 $$\Delta E_{\mathrm{int}}=E_{P:L}-E_{P}-E_{L}$$

by applying a consistent MBE­(*n*) approximation to each of *E*_P_, *E*_L_, and *E*_P:L_. Many terms cancel *a priori* in the difference *E*_P:L_ – *E*_P_, because Δ*E*_*AB*···_ does not contribute to Δ*E*_int_ if all fragments *AB*··· reside within the protein.^19^ This simplification is handled automatically within Fragme∩t,^19,31^ and it affords dramatic savings as compared to naive application of MBE­(*n*) to eq 4. For the two P:L complexes in Figure 1, elimination of these unnecessary terms reduces the total compute cost by up to 97% for MBE(2) calculations at a semiempirical level of theory,^6^ because the only terms Δ*E*_*AB*_ that are required are those where *B* is the ligand.

As in previous work,^6,7,33^ we use fragments consisting of single amino acids for the protein. The ligand is not fragmented in these calculations, which increases the cost but serves to establish a baseline for convergence of Δ*E*_int_. Ligand fragmentation can be considered at a later time, perhaps using tokenization strategies developed for fragment-based drug design.^34–37^

In previous applications of MBE­(*n*) to enzymatic systems, we focused on active-site models with *N* = 30–35 residues, selected based on distance from the substrate,^33^ and we have also used distance thresholds to construct binding-site models for Δ*E*_int_(P:L) calculations.^7^ However, the metalloenzymes examined here are significantly larger (*N* ≥ 160 fragments), with sizable ligands (Figure 1). This necessitates a “bottom-up” graph-theoretical screening approach to make these calculations feasible.^23,31^ This approach is described next and is reported here for the first time in protein systems.

### 2.2 Screening

Fragme∩t represents a fragmentation scheme in the form of a directed acyclic graph that specifies parent/child relationships between subsystems.^23,31^ Each child is the union of its parents; for example, the trimer *ABC* is the child of dimers *AB*, *AC*, and *BC*. The graph is constructed in layers starting with monomer terms followed by dimers, trimers, etc., up to a user-defined terminal order or until no new subsystems can be added due to lack of eligible parents.

As nodes are eliminated from the graph at each *n*-body order, due to energy and/or distance screening, a given child may be missing one or more of its parents (Figure 2). Let *m* denote the number of missing parents. Previous work on water clusters, using single-H_2_O fragments, suggests that subsystems with *m* > 1 make negligible contribution and can be omitted *a priori*.^23^ For those systems, however, the *m* = 1 terms contribute significantly and accuracy is degraded if a more aggressive *m* > 0 screening criterion is applied.

**Figure 2:**
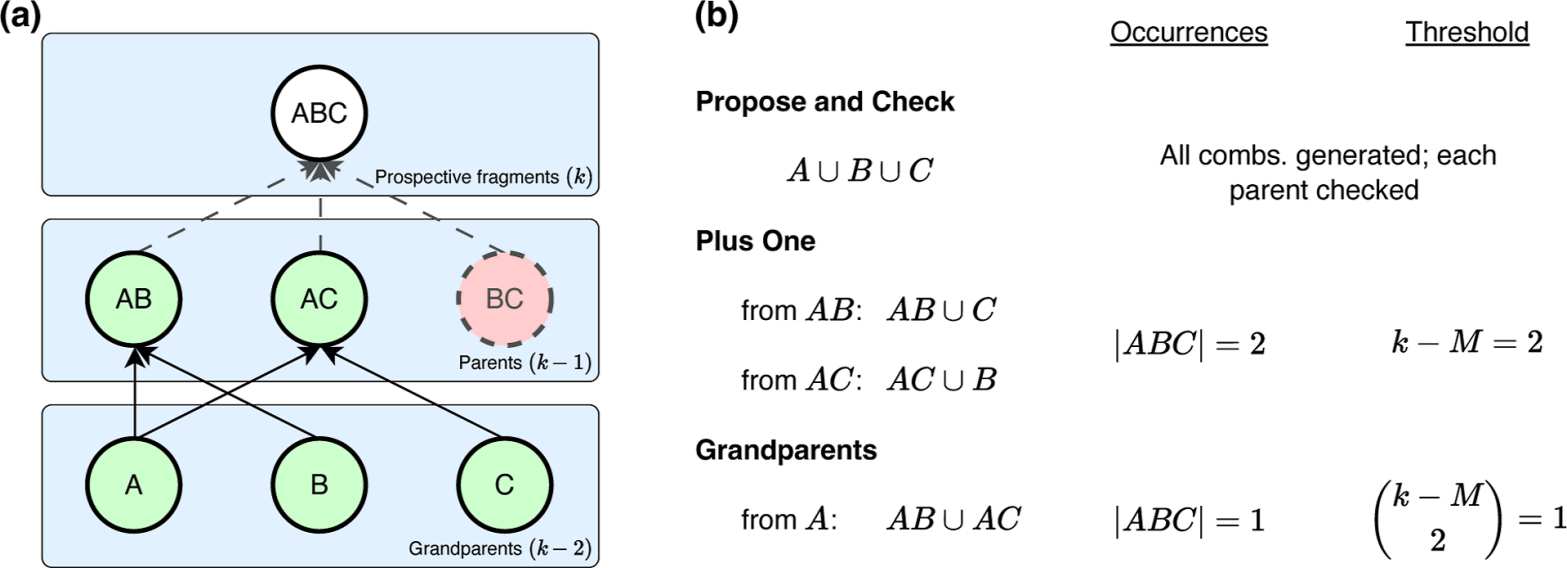
Illustration of bottom-up construction of the many-body interaction graph, applied to a hypothetical system consisting of monomers {*A*, *B*, *C*}. (a) Representation of the subsystems as a directed acyclic graph. Nodes *AB* and *AC* (in green) have survived the two-body screening process and are considered as parents for the trimer layer, whereas *BC* (highlighted in red) failed the screening test and was eliminated. (b) Three possible ways to implement a screening algorithm, as described in the text. All three algorithms afford the same result but their efficiencies differ.

We define a screening parameter *M* indicating the maximum number of missing parents that is allowed, so that nodes on the graph with *m* > *M* are deleted. For the two metalloenzymes considered here, we observe minimal increase in accuracy (∼0.1 kcal/mol) for *M* = 0 versus *M* = 1; see Figure S1. Testing an even more conservative *M* = 2 procedure is prohibitively expensive, adding almost 9000 subsystems for MBE(3) calculations on 1ZP5. However, the negligible difference between *M* = 0 and *M* = 1 results suggests that the latter is sufficient to achieve convergence.

In the present work, we use *M* = 1 as an initial screening criterion at each *n*-body order, prior to the application of any distance or energy cutoffs.^31^ Several possible algorithms for performing this initial screening are discussed in Section 2.2.1. Energy and distance screening are then described in Section 2.2.2.

#### 2.2.1 Screening the Parent/Child Relationships

Eliminating nodes on the many-body interaction graph is crucial for obtaining the sparsity that makes higher-order MBE­(*n*) calculations tractable.^23,31^ Even with a fast semiempirical QC method, energy-based screening becomes prohibitively expansive for *n* ≥ 4 unless the interaction graph is first culled based on the number of missing parents. In previous work,^23^ the requisite parental checks were performed at many-body order *k* by considering all

5 (NPk)=NP!k!(NP−k)!

potential new subsystems, where *N*_*P*_ is the number of primary fragments. Each proposed subsystem was checked to determine whether *k* – *M* parents were present in the graph. We refer to this as the *propose-and-check* algorithm. In the hypothetical example of Figure 2a, we propose adding *ABC* to the graph at order *k* = 3. Finding that this subsystem has one missing parent (*m* = 1), it is accepted and added as a node in the trimer layer of the graph.

The performance of the propose-and-check algorithm scales poorly with the number of fragments, as illustrated in Figure 3 for (H_2_O)_*N*_ clusters with *N* = 6–55 monomers. The cost scales as $O\left(N_{P}^{k}\right)$ albeit with a dramatically smaller prefactor as compared to performing a semiempirical energy evaluation on each *k*-mer of fragments. This makes it feasible to extend (H_2_O)_64_ calculations to MBE(8) even though there are formally ∼4×10^9^ unique octamers. For an enzyme with *N* = 160 monomers, however, this is almost 9 × 10^12^ terms at *n* = 8, which is untenable.

**Figure 3:**
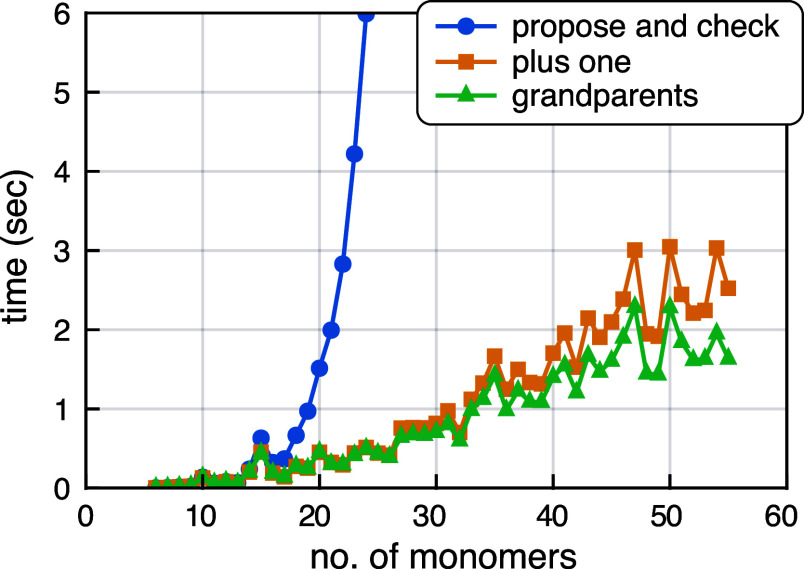
Performance of bottom-up fragmentation using the algorithms described in Figure 2. Benchmarks are reported for (H_2_O)_*n*_ clusters (*N*= 6–55) with distance cutoffs *d*_2_ = 6 Å and *d*_3_ = 5 Å. The MBE­(*n*) expansions were allowed to progress up to *n* = 8.

Thus, for the present work the bottom-up algorithm was further optimized beyond the simple propose-and-check strategy. To see how, observe that each child differs from its parents by addition of a single monomer (Figure 2a). A *plus-one* algorithm attempts to add each monomer to each prospective parent. In the three-body example of Figure 2a, this means adding *A*, *B*, or *C* to either of *AB* or *BC*, since *AC* has been eliminated. More generally, to add *k*-body subsystems one must consider *S*_*P*_ × *S*_*k*–1_ where *S*_*P*_ is the set of primary monomers and *S*_*k*–1_ is the set of (*k* – 1)-body parents that have not been eliminated from the graph. The occurrences of each unique subsystem are counted and those occurring at least *k* – *M* times are proposed for addition to the graph, subject to further energy or distance screening.

In the example of Figure 2a, prospective fragment *ABC* manifests twice during this process, which meets our threshold of required parents: *k* – *M* = 2 when *M* = 1 missing parent is allowed. As compared to the propose-and-check algorithm, this plus-one approach better leverages sparsity of the many-body interaction graph, avoiding unnecessary parental oversight. This is reflected in the timing data shown in Figure 3.

The plus-one algorithm can be further optimized by considering the grandparents of prospective fragments. This *grandparents* algorithm (Figure 2b) starts by considering the prospective grandparents, which are *A*, *B*, and *C* for the trimers in Figure 2a. When adding a new generation of subsystems at order *k*, the pairwise unions of the children of all grandparents (at order *k* – 2) are formed and the unique occurrences are counted. Fragments that occur at least (*k* – *M*)­(*k* – *M* – 1)/2 times are proposed for addition to the graph. In the example of Figure 2a the trimer *ABC* occurs only once, via *AB* ∪ *AC*, which meets the threshold with *k* = 3 and *M* = 1. In that example, grandparents *B* and *C* have only a single child each so they do not contribute to the count.

Timings for this algorithm are also shown in Figure 3 and it offers a modest improvement relative to the plus-one algorithm. In what follows, the grandparents algorithm is used wherever feasible. As its name implies, it requires there to be (*k* – 2)-order fragments already in the graph, so the grandparents algorithm is only suitable for three-body and higher-order interactions, and only when *k* – *M* > 1 where *M* is the number of missing parents that are allowed. If these conditions are not satisfied, then the plus-one algorithm is used instead.

#### 2.2.2 Energy and Distance Screening

At a given *n*-body order, energy and/or distance screening is applied following the missing-parents screening. For example, at the *n* = 3 level we eliminate the trimer *ABC* if

6 $$\left|\Delta E_{ABC}^{low}\right|<\tau_{3B}$$

where Δ*E*_*ABC*_^low^ is the three-body correction evaluated at a low level of theory and τ_3B_ is a user-specified threshold.

In addition, we might also examine

7 $$R_{ABC}=\max\left\{R_{AB},R_{BC},R_{AC}\right\}$$

for some suitable definition of the interfragment distances, say

8 $$R_{AB}=\min_{a\in A,b\in B}∥r_{a}-r_{b}∥$$

Three-body distance screening would proceed by comparing *R*_*ABC*_ in eq 7 against a user-specified threshold *d*_3_, eliminating *ABC* if

9 $$R_{ABC}<d_{3}$$

### 2.3 Computational Methods

Structure preparation closely follows our previous work,^7^ but for completeness the protocol is described below along with other details of the procedure.

#### 2.3.1 Structure Preparation and Setup

Crystal structures from the protein data bank (PDB) were protonated using the H++ web server (pH = 7.0, salinity = 0.15 M, ε_in_ = 10, and ε_out_ = 80).^38^ Ligands were protonated separately using PyMOL.^39^ The resulting P:L structures were then relaxed using GFN-FF,^40^ a polarizable force field designed for biological macromolecules, in conjunction with a generalized Born/solvent-accessible surface area (GB/SASA) implicit solvation model, representing water.^41^ These relaxed structures are available in the Supporting Information. The 1MMQ structure is the same as that reported previously,^7^ but the 1ZP5 structure includes some additional relaxation (using GFN2-xTB) to ensure correct protonation states of the histidine residues located nearest to each metal ion. Following structure relaxation, most crystallographic water molecules were removed except for those that were directly coordinated to the ligand or to ionic moieties.

#### 2.3.2 Fragmentation

As in previous work,^6,7,33^ fragments consist of single amino acid residues, cutting the C_α_–C­(=O) carbon–carbon bond and capping with hydrogen atoms. Note that even the “molecular fractionation with conjugated caps” (MFCC) method for P:L interaction energies is nowadays used with hydrogen-atom caps (albeit with overlapping amino acid fragments),^42^ suggesting that more sophisticated capping strategies are unnecessary. The ligand was considered as a single fragment. Ionic cofactors were combined into a monomer with their nearest residues (within 2.5 Å), in order to improve the stability of MBE­(*n*) calculations and to reduce the number of fragments. However, both 1MMQ and 1ZP5 contain a divalent ion within 2.5 Å of the ligand that cannot be combined in this way, because it must be separated from the ligand in order to compute Δ*E*_int_.

Energy-based screening was performed using the bottom-up approach described in Section 2.2, allow for *M* = 1 missing parent. This marks the first time that this procedure has been applied to biomolecular systems, so we tested a range of two- and three-body energy thresholds τ_2B_ and τ_3B_, respectively, defined in the sense of eq 6 and the analogous two-body expression

10 $$\left|\Delta E_{AB}^{low}\right|<\tau_{2B}$$

We use the semiempirical HF-3c model^43^ for the low-level screening. This marks a change from previous work on water clusters,^23^ where we used the GFN2-xTB tight-binding model^44^ for this purpose. For subsystems with net charge, we find that GFN2-xTB often exhibits convergence problems whereas HF-3c is more robust. Two-body energy screening was performed using thresholds τ_2B_ in the range 0.05–1.00 kcal/mol, while three-body body screening was performed with a consistent threshold τ_3B_ = 0.05 kcal/mol.

A distance threshold was used to further reduce the number of systems in some cases, which will be indicated explicitly. The distance between two subsystems is defined as the minimum interatomic distance between any two atoms (*R*_*AB*_ in eq 8), and dimers are eliminated if *R*_*AB*_ < *d*_2_ = 8 Å. This is the same distance cutoff used in previous work.^6,7,33^

#### 2.3.3 QC Calculations

All calculations were performed using Fragme∩t,^23^ interfaced to Q-Chem^32^ (v. 6.3) for the QC calculations. For timing data, calculations were run on 48-processor nodes (Dell PowerEdge C6620 two-socket server), using four worker processes per node, with each individual Q-Chem calculation employing four threads. Supersystem calculations, used to obtain benchmarks for MBE­(*n*) approximations, were performed using a single 48-core node (Intel Xeon Platinum 8268). Timings are reported in terms of total central processing unit (CPU) time, aggregated across all processors, rather than “wall time”, because aggregate CPU time is the more appropriate metric for evaluating the true cost of fragment-based QC calculations.^15,45^ The self-consistent field convergence criterion was set to 10^–8^ *E*_*h*_ for all calculations. Integral screening and shell-pair drop tolerances were set to 10^–12^ a.u., consistent with recommendations for large-molecule calculations using diffuse basis sets.^46^

The HF-3c model is used for energy screening but we also report some full-protein Δ*E*_int_(P:L) values at this level of theory. In addition, DFT calculations are performed using the ωB97X-V functional,^47^ which performs well for noncovalent interactions.^47,48^ Whereas HF-3c uses a specialized minimal basis set,^43^ for the DFT calculations we employ minimally augmented versions^49^ of the Karlsruhe augmented basis sets.^50^ These are denoted def2-ma-SVP, def2-ma-TZVP, and def2-ma-QZVP.^49^ Diffuse functions can be important for noncovalent interaction energies but minimal augmentation is typically sufficient for DFT.^14^

All QC calculations are performed using dielectric boundary conditions with a dielectric constant ε = 4, implemented via the conductor-like polarizable continuum model (C-PCM).^51,52^ The value ε = 4 is appropriate for the hydrophobic interior of a protein,^53–58^ but at the same time the use of low-dielectric boundaries mitigates spurious oscillations in the MBE­(*n*) sequence of approximations.^19,20,33^ Oscillations with respect to the *n*-body order are especially problematic in the presence of ionic side chains^33^ and are driven by self-interaction error that is amplified by fragmentation.^19,20^ Even low-dielectric boundaries can provide a driving force for charge localization, however.^20,59,60^ (The same effect can be accomplished with embedding charges.^61^) The solute cavity for C-PCM calculations was constructed using Bondi’s atomic radii,^62^ scaled by 1.2, which is a standard “van der Waals” cavity construction.^52^ This interface was discretized using the switching/Gaussian procedure,^63–65^ with 50 Lebedev points for hydrogen and 110 points for other nuclei. For larger supersystem calculations of the entire protein, a conjugate gradient implementation of C-PCM was used.^65^

## 3 Results and Discussion

As in previous work,^6,7,33^ we begin by using HF-3c to compute Δ*E*_int_ for the unfragmented P:L complex. Using this full-protein baseline, we can then estimate the error introduced by the fragmentation approximation and test the convergence of the screening and *n*-body approximations (Section 3.1). Results obtained with DFT calculations are discussed subsequently (Section 3.2). Raw data for the plots that follow can be found in the Supporting Information.

### 3.1 HF-3c Calculations

For 1MMQ, the structure is unchanged from previous work.^7^ Thus, the HF-3c result is the same: Δ*E*_int_ = – 178.6 kcal/mol, requiring 5619 CPU hours on 48 processors. In contrast, |Δ*E*_int_| for 1ZP5 is smaller than previously estimated due to additional structural relaxation in the present work: Δ*E*_int_ = – 76.7 kcal/mol, requiring 3138 CPU hours on the same hardware.

#### 3.1.1 Convergence Tests

We first examine convergence of MBE­(*n*) calculations at the HF-3c level for these supramolecular P:L benchmarks, with results for 1MMQ shown in Figure 4 and those for 1ZP5 in Figure 5. Both plots have the same structure and Figure 4a, for example, plots absolute errors in the fragmentation approximation relative to a supramolecular calculation at the same level of theory, using MBE­(*n*) approximations up to *n* = 7. Results are juxtaposed for several different values of the two-body screening threshold (τ_2B_), using a fixed value τ_3B_ = 0.05 kcal/mol that has worked well for water clusters.^23^ (Results for other values of τ_3B_ can be found in Tables S1 and S2). All calculations set *M* = 1 as the maximum number of missing parents, as described in Section 2.2. In addition, a pairwise distance cutoff *d*_2_ = 8 Å is used in some calculations. This particular cutoff value is a conservative choice that we have used in previous MBE­(*n*) calculations targeting enzymatic thermochemistry.^6,7,33^

**Figure 4:**
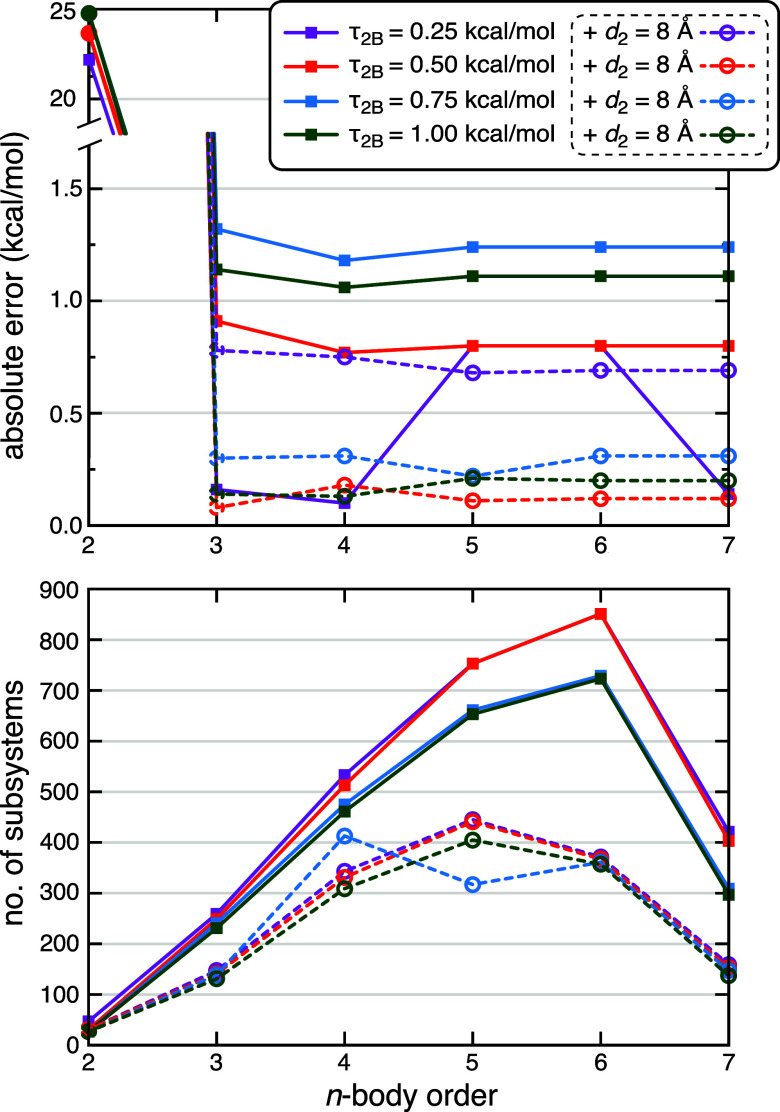
Convergence of MBE­(*n*) calculations at the HF-3c level for Δ*E*_int_(P:L) in 1MMQ: (a) absolute errors versus a full-protein benchmark at the same level of theory, and (b) number of distinct subsystem QC calculations required. Results are shown for color-coded values of τ_2B_, using either energy screening alone (solid lines and square symbols) or else a combination of energy screening with a distance cutoff *d*_2_ = 8 Å (dashed lines and round symbols). All calculations use τ_3B_ = 0.05 kcal/mol and *M* = 1 to screen the many-body interaction graph. The energy scale in (a) is broken to illustrate the very large errors (up to 25 kcal/mol) at the two-body level, which drop below 1.5 kcal/mol for *n* ≥ 3.

**Figure 5:**
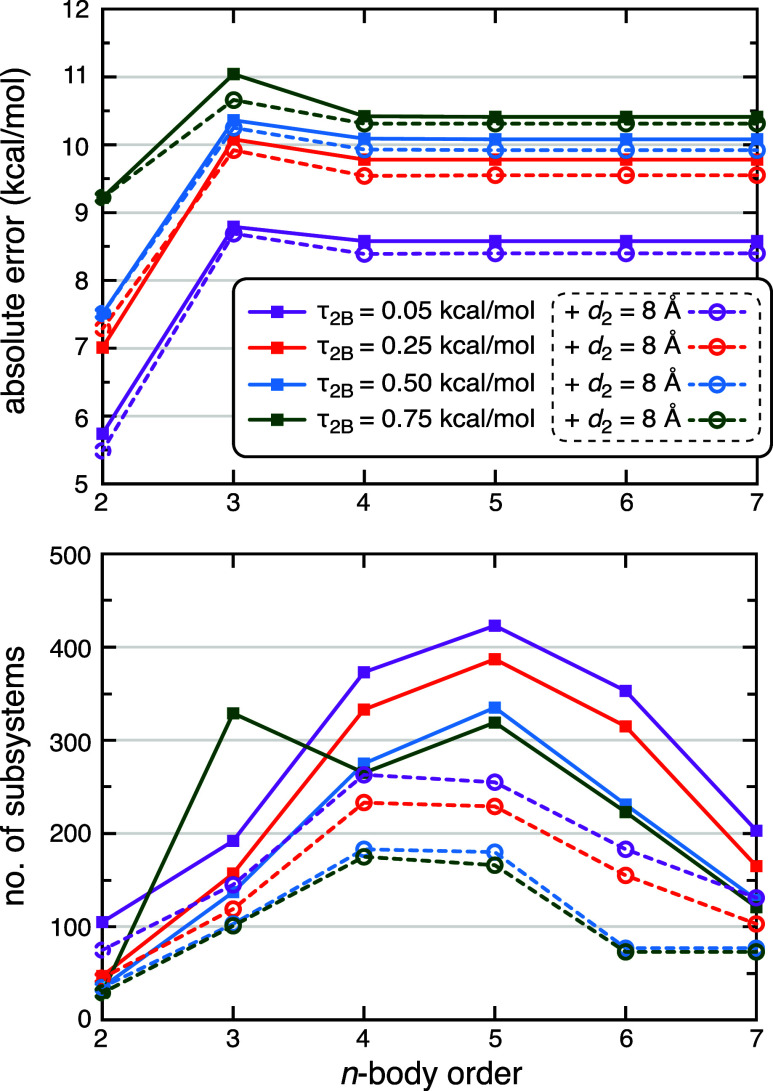
Convergence of MBE­(*n*) calculations at the HF-3c level for Δ*E*_int_(P:L) in 1ZP5: (a) absolute errors versus a full-protein benchmark at the same level of theory, and (b) number of distinct subsystem QC calculations required. Results are shown for color-coded values of τ_2B_, using either energy screening alone (solid lines and square symbols) or else a combination of energy screening with a distance cutoff *d*_2_ = 8 Å (dashed lines and round symbols). All calculations use τ_3B_ = 0.05 kcal/mol and *M* = 1 to screen the many-body interaction graph.

The number of unique subsystems, which is the number of distinct QC calculations that is required (following screening), is plotted for 1MMQ in Figure 4b at each *n*-body order, and for 1ZP5 in Figure 5b. We find that the combination of energy and distance screening can reduce the number of target-level QC calculations by ∼50% as compared to energy screening alone, without much effect on Δ*E*_int_ (typically <1 kcal/mol change). In other words, only residues within 8 Å of the ligand need to be considered. While that number might be expected to be somewhat system-dependent, the presence of several divalent ions in these metalloenzymes likely makes this a fairly conservative estimate for application to other P:L complexes.

Applying *d*_2_ = 8 Å to the 1MMQ system engenders a small reduction in error as compared to the case where no distance threshold is applied (Figure 4a), except for the most conservative value of τ_2B_. This may simply be error cancellation, as the errors are already small and the same effect is not consistently observed for the tight threshold τ_2B_ = 0.25 kcal/mol. On the other hand, distance screening substantially reduces the number of subsystems (Figure 4b) and may result in a more robust *n*-body expansion. Distance screening has a much smaller effect on Δ*E*_int_ for 1ZP5 (Figure 5a), for which the number of subsystems is ≲ 400 even without distance screening (Figure 5b). This is comparable to the number of subsystems in 1MMQ once *d*_2_ = 8 Å is applied, supporting the idea of minor stability problems when the number of subsystems is larger that ∼400.

A curious effect is that use of higher-order *n*-body expansions can actually *reduce* the total number of subsystems.^23,31^ This effect is explained carefully in ref 31. Briefly, if one imagines inserting formulas for Δ*E*_*AB*_ (eq 2) and Δ*E*_*ABC*_ (eq 3) into the MBE(3) formula (eq 1), the result would be a large number of individual terms with numerous redundancies. These can be eliminated by analytic resummation, affording only the unique subsystems multiplied by combinatorial coefficients.^16,66^ Computationally, Fragme∩t’s database and cryptographic hash architecture accumulates these coefficients (in a manner that is more efficient than other competing software architectures),^31^ ensuring that only unique QC calculations are performed. These are subsequently multiplied by an appropriate combinatorial factor. Thus, numerous lower-order terms are subsumed into higher-order corrections as the *n*-body order increases. The result is a decrease in the number of distinct QC calculations, although the subsystem calculations themselves become larger and more expensive.

A main takeaway from the error convergence plots in Figures 4a and 5a is that it is possible to converge these Δ*E*_int_(P:L) calculations at relatively low *n*-body orders, arguably at *n* = 3 (for 1MMQ) and certainly by *n* = 4 (for 1ZP5). These MBE­(*n*) calculations require only a few hundred subsystems. The new screening procedure allows us to demonstrate convergence unambiguously, by extending the calculations all the way to *n* = 7. For the 1MMQ complex the converged errors are <1.5 kcal/mol, which is <1% of Δ*E*_int_.

The 1ZP5 complex, on the other hand, exhibits greater sensitivity with respect to τ_2B_ and also manifests a systematic error that does not converge to zero as *n* increases. A residual error of 8–10 kcal/mol remains, depending somewhat on the value of τ_2B_, and does not disappear when τ_3B_ is reduced by a factor of 2 (Table S2). We do not have an explanation for this residual error, which we plan to investigate in future work including the use of larger, overlapping fragments, as in recent MFCC calculations of P:L interactions.^42^ That said, the residual error is no larger than 0.07 kcal/mol/fragment. It has been argued that fragmentation errors smaller than 0.1 kcal/mol/fragment are ignorable,^7,24,31^ because that value represents 10% of *k*_B_ *T* (per fragment) at room temperature. Electronic structure errors at that level cannot be distinguished from thermal fluctuations.

For 1ZP5, we observe a significant difference in the magnitude of Δ*E*_int_ as compared to earlier MBE(3) results at the HF-3c level. Partly this is due to better convergence of the present calculations but may also be partially attributable to structural differences between the 1ZP5 models. Here, positions of the protons coordinated to the Zn^2+^ ion nearest the ligand have been adjusted, which in turn affects the hydrogen-bonding network involving the ligand, a water molecule, and the E119 residue. This highlights the critical impact of both structure preparation and also fluctuations, which can have a large impact on thermochemical calculations.^67^ For this reason, others have emphasized that the use of QC in drug design does not obviate the requirement to perform sampling.^68^ QC calculations often converge in 50–100 snapshots along a molecular dynamics trajectory for a single binding pose,^69,70^ which is certainly feasible with protocols developed here but lies beyond the scope of the present work.

#### 3.1.2 Analysis of Many-Body Terms

Using energy-based screening, we achieve errors per monomer that are nearly equivalent to what we reported previously using only distance-based screening,^7^ yet the present calculations incur only a fraction of the computational cost. Distributions of *n*-body corrections Δ*E*_*AB*···_ are plotted in Figure 6 for both complexes and the aggregate *n*-body contributions are provided in Table S3. The most significant contributions to Δ*E*_int_ arise from the two- and three-body terms with a modest contribution from the four-body terms, suggesting that the convergence observed above is not accidental.

**Figure 6:**
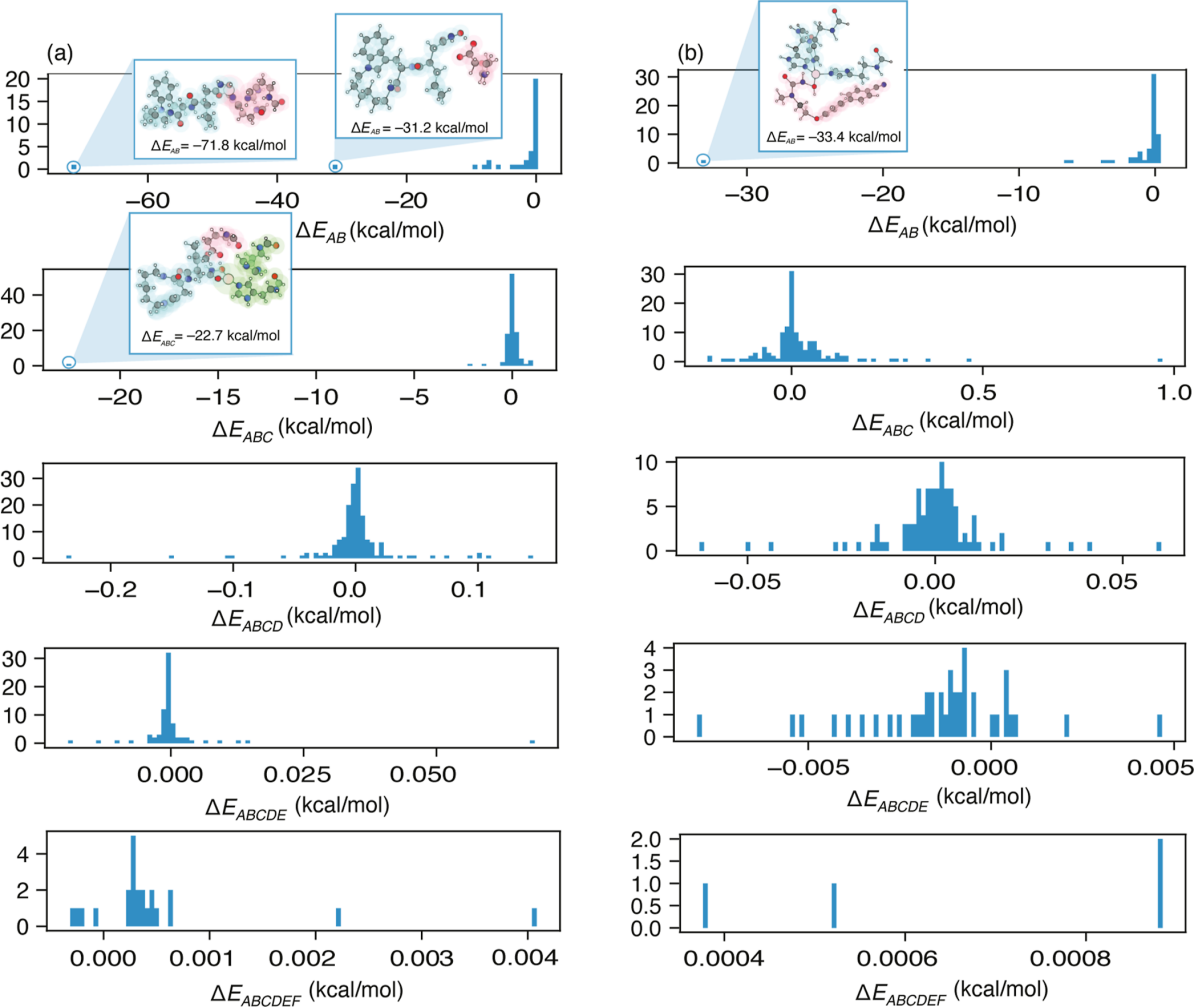
Distribution of MBE­(*n*) terms contributing to Δ*E*_int_ for (a) 1MMQ and (b) 1ZP5 at the HF-3c level of theory. Various *n*-body contributions are arranged from the top (two-body corrections Δ*E*_*AB*_) to the bottom (six-body corrections Δ*E*_*ABCDEF*_). These histograms include all terms that are not screened out by the combination of parameters τ_2B_ = 0.25 kcal/mol, τ_3B_ = 0.05 kcal/mol, and *M* = 1 for 1MMQ and τ_2B_ = 0.05 kcal/mol, τ_3B_ = 0.05 kcal/mol, and *M* = 1 for 1ZP5. Two- and three-body corrections larger than 20 kcal/mol in magnitude are highlighted. See Table S3 for a tabulation of the net *n*-body contributions.

For 1MMQ, the largest contributions to Δ*E*_int_ arise from interactions between the ligand and either the nearby Zn^2+^ ion or the hydrogen-bonded glutamate residue, the latter of which has a +1 charge. A 24 kcal/mol reduction in the error between *n* = 2 and *n* = 3 (Figure 4a) results from inclusion of a trimer consisting of all three moieties, with only marginal contributions from other trimers. These strongly interacting dimers and trimer are shown explicitly in Figure 6a.

In contrast, 1ZP5 has only a single large interaction term, between the ligand and a nearby Zn^2+^ ion (Figure 6b). Nevertheless, there is a significant difference in the accuracy of the MBE(2) and MBE(3) approximations (Figure 5a). This arises from the aggregate effect of numerous three-body terms, none of which is overwhelmingly larger than the others. Unlike 1MMQ, where the importance of the ligand–glumate–Zn^2+^ trimer was probably identifiable *a priori*, this example requires a systematic, automatable approach to identify the important interactions.

### 3.2 DFT Interaction Energies

HF-3c results demonstrate convergence behavior but may not afford reliable interaction energies, although we have argued that HF-3c values for Δ*E*_int_(P:L) may be good enough for some practical purposes if combined with sampling over geometries.^7^

In any case, we wish to examine the use of screened MBE­(*n*) approximations to obtain DFT interaction energies in large binding-site models. Here, one must additionally consider convergence with respect to the choice of basis set. Tables 1 and 2 present DFT results for the two metalloenzymes using the ωB97X-V functional and basis sets ranging from double-ζ to quadruple-ζ. All calculations employ energy screening with HF-3c, and we also consider additional distance screening with *d*_2_ = 8 Å.

**Table 1:** ωB97X-V Results for 1MMQ Using Energy (+Distance) Screening

		no. subsystems		Δ*E*_int_ (kcal/mol)		CPU time (h)	
basis set^a^	MBE (*n*) order	*E*-only^b^	Δ(*E* + *R*)^c^	*E*-only^b^	Δ(*E* + *R*)^c^	*E*-only^b^	Δ(*E* + *R*)^c^
DZ	2	31	(+0)	–129.5	(+0.00)	40	(+0)
DZ	3	249	(−108)	–147.7	(−1.2)	509	(−182)
DZ	4	513	(−182)	–148.0	(−1.3)	1876	(−635)
DZ	5	753	(−312)	–148.4	(−1.1)	4220	(−1723)
DZ	6	851	(−484)	–148.3	(−1.2)	6236	(−3430)
DZ	7	403	(−250)	–148.3	(−1.2)	4124	(−2900)
TZ	2	31	(+0)	–116.2	(+0.0)	182	(+0)
TZ	3	249	(−108)	–135.8	(−1.4)	2250	(−761)
TZ	4	513	(−182)	–135.5	(−1.4)	8528	(−2813)
TZ	5	753	(−312)	–135.8	(−1.2)	18,878	(−7515)
TZ	6	851	(−484)	–135.7	(−1.3)	27,142	(−14,592)
TZ	7	403	(−250)	–135.7	(−1.2)	15,935	(−10,655)
QZ	2	31	(+0)	–115.7	(+0.0)	1992	(+0)
QZ	3	249	(−108)	–135.9	(−1.4)	24,095	(−8191)

a DZ = def2-ma-SVP, TZ = def2-ma-TZVP, QZ = def2-ma-QZVP.

b Result obtained using energy screening only, with parameters τ_2B_ = 0.5 kcal/mol, τ_3B_ = 0.05 kcal/mol, and *M* = 1.

c Change in the energy-screening result when distance screening with *d*_2_ = 8 Å is added.

**Table 2:** ωB97X-V Results for 1ZP5 Using Energy (+Distance) Screening^b^ ^c^

		no. subsystems		Δ*E*_int_ (kcal/mol)		CPU time (h)	
basis set^a^	MBE(*n*) order	*E*-only^a^	Δ(*E* + *R*)^a^	*E*-only^a^	Δ(*E* + *R*)^a^	*E*-only^a^	Δ(*E* + *R*)^a^
DZ	2	35	(+0)	–74.5	(+0.0)	28	(+0)
DZ	3	137	(−34)	–75.1	(+0.1)	265	(−60)
DZ	4	275	(−92)	–76.5	(+0.1)	1093	(−402)
DZ	5	335	(−155)	–77.0	(+0.1)	1836	(−841)
DZ	6	231	(−154)	–77.0	(+0.1)	1340	(−907)
DZ	7	129	(−52)	–77.0	(+0.1)	822	(−389)
TZ	2	35	(+0)	–60.7	(+0.0)	135	(+0)
TZ	3	137	(−34)	–59.9	(+0.1)	1315	(−1159)
TZ	4	275	(−92)	–61.0	(+0.1)	5588	(−2047)
TZ	5	335	(−155)	–61.2	(+0.2)	9609	(−8811)
TZ	6	231	(−154)	–61.1	(+0.2)	7146	(−4804)
TZ	7	129	(−52)	–61.1	(+0.2)	4397	(−2055)
QZ	2	35	(+0)	–58.6	(+0.0)	1421	(+0)
QZ	3	137	(−34)	–58.1	(+0.1)	13,611	(−2971)

a DZ = def2-ma-SVP, TZ = def2-ma-TZVP, QZ = def2-ma-QZVP.

b Result obtained using energy screening only, with parameters τ_2B_ = 0.5 kcal/mol, τ_3B_ = 0.05 kcal/mol, and *M* = 1.

c Change in the energy-screening result when distance screening with *d*_2_ = 8 Å is added.

A possible concern is that minimal-basis HF-3c calculations might converge at a lower *n*-body order as compared to DFT calculations that incorporate a more complete description of polarization, but this is not observed in practice. For both P:L complexes, the DFT-MBE(3) value of Δ*E*_int_ lies within 2 kcal/mol of the DFT-MBE(7) result in a given basis set, and the DFT-MBE(4) value lies within 0.5 kcal/mol of the *n* = 7 result. These observations are valid for both the double- and triple-ζ basis sets; the quadruple-ζ results extend only to *n* = 3, for reasons of cost. To some extent, that high cost is an artifact of our decision not to fragment the ligand and doing so might allow higher-order *n*-body calculations at the basis-set limit although it is not clear what else we would learn about convergence behavior. Many-body counterpoise corrections,^73,74^ which are being added to the Fragme∩t code, are likely to render quadruple-ζ calculations unnecessary.^14^

Although *n*-body convergence behavior is insensitive to the choice of basis set (including minimal basis sets when considering the HF-3c results), the numerical value of Δ*E*_int_ certainly depends on the basis set. Differences between converged MBE­(*n*) interaction energies in double- versus triple-ζ basis sets are 12 kcal/mol for 1MMQ and 15 kcal/mol for 1ZP5. Quadruple-ζ results at the two- and three-body level suggest that the triple-ζ results may be within ∼1 kcal/mol of the basis-set limit, which is consistent with other large-scale DFT results for P:L interaction energies.^14^

While energy screening alone facilitates convergence of these DFT-MBE­(*n*) results, both with respect to *n* and with regard to basis set, the addition of a conservative 8 Å distance cutoff significantly accelerates the calculations. Tables 1 and 2 quantify this in terms of how the cutoff reduces the number of distinct subsystem calculations, modifies Δ*E*_int_(P:L), and reduces the aggregate CPU time. With regard to Δ*E*_int_(P:L), the 8 Å cutoff changes the result by only about 1.2 kcal/mol for 1MMQ and by ∼0.1 kcal/mol for 1ZP5, while reducing the cost by hundreds or thousands of CPU hours. In some cases, this is a 50% reduction in CPU time as compared to the use of energy screening alone.

This is worth considering with an eye toward eventual sampling over geometries. First, note that P:L interaction energies |Δ*E*_int_| computed using QC are generally at least an order of magnitude larger than thermodynamic binding free energies, $\left|\Delta G_{bind}^{⊖}\right|$ .^7,69,70^ For the present examples, Δ*E*_int_(1MMQ) ≈ – 136 kcal/mol whereas experimental estimates of the binding affinity Δ*G*_bind_^⊖^(1MMQ) range from −10.6 kcal/mol^26,71^ to −11.6 kcal/mol.^71,72^ Likewise, Δ*G*_bind_^⊖^(1ZP5) = –4.0 kcal/mol^71,72^ is much smaller than the interaction energy obtained here, Δ*E*_int_(1ZP5) ≈ – 61 kcal/mol. These disparities persists even when Δ*E*_int_ is corrected for the effects of thermal averaging, to obtain an ensemble-averaged value ⟨Δ*E*_int_⟩. Thermal fluctuations in Δ*E*_int_ may be ∼2 kcal/mol or larger under ambient conditions,^69,70^ exceeding the convergence errors that are documented here, and entropic corrections are considerably larger still. Nevertheless, correlations between single-pose QC values of Δ*E*_int_ and experimental binding affinities are sometimes found to be reasonable, even without careful consideration of convergence.^27–29,75–77^ In short, the ∼1 kcal/mol convergence targeted here is probably overly conservative. However, the present work does provide a testable means to evaluate convergence, should questions or discrepancies arise.

Along those lines and to contextualize the improvement offered by the combined energy- and distance-based screening approach, we compare the current results for 1MMQ with previous results based on MBE­(*n*) with only distance screening.^7^ For an identical geometry of the P:L complex, the distance-only approach affords Δ*E*_int_ values that differ from those reported here using the composite screening approach, by 3 kcal/mol for MBE(2) and by 7 kcal/mol for MBE(3). However, the older set of calculations were limited to lower-order *n*-body terms due to proliferation of subsystems in the absence of energy screening. Thus, the composite approach is more reliable for establishing the QC value of Δ*E*_int_ in the absence of a supramolecular (full-protein) benchmark. The cost is also substantially reduced. For 1MMQ, a DFT-MBE(3) calculation at the ωB97X-V/def2-ma-TZVP level required 62,962 CPU hours as compared to 1489 h for the analogous calculation with energy and distance screening, a 97% decrease in aggregate computing time.

## 4 Conclusions

Accurate modeling of P:L interactions using fragment-based QC presents a challenge insofar as long-range decay of electrostatic interactions may render distance-based fragmentation protocols inefficient or inaccurate. The problem is expected to be more severe in the presence of divalent metal cations,^21^ as in the examples considered here. Nevertheless, a hybrid screening protocol combining energy-based selection with a conservative distance cutoff overcomes these limitations.

The pivotal modification driving this success is an energy-based filter for *n*-body subsystems. Distance-based screening, as in our previous approach to computing P:L interactions in these same metalloenzymes,^6,7^ relies on cutoffs that indiscriminately incorporate a large number of negligible interactions yet miss others that are distant but electrostatically significant. By evaluating the various *n*-body corrections at a low-cost semiempirical level of theory, we retain only those terms that contribute significantly to the total energy, bypassing the expensive but physically irrelevant combinatorial proliferation of subsystems. This represents a fundamental paradigm shift toward tractability, compared to previous methods where truncation at MBE(3) or MBE(4) was mandatory and convergence behavior was unclear. By means of energy screening, we are able to extend the MBE­(*n*) calculations to *n* = 7 and demonstrate unambiguously that results converge at the four-body level.

The addition of a conservative 8 Å distance cutoff on top of this procedure does not fundamentally alter the convergence properties but further reduces the cost, enabling DFT-MBE­(*n*) calculations to be extended to triple-ζ basis sets with diffuse functions. Calculations of at least DFT/triple-ζ quality are crucial to establishing accurate benchmarks for noncovalent interaction energies in large systems.^14^ This methodology paves the way for high-throughput, high-accuracy QC calculations as a means to generate training and/or assessment data for machine learning and other low-cost methods for P:L interactions.

## Supplementary Material









## Notes

Processed data tables (PDF)
Raw data (XLSX)
Coordinates for the relaxed structural models (PDB)
(PDB)
