# Supplementary material for: Energy-Screened Many-Body Expansion for Protein–Ligand Interactions: Examining Convergence for Metalloenzymes Through Seven–Body Interactions
Source: J Chem Theory Comput. 2026 Mar 30;22(7):3720–31. doi: 10.1021/acs.jctc.6c00190 (PMC13085233; doi:10.1021/acs.jctc.6c00190)
Supplement: Supplementary file 1 [file ct6c00190_si_001.pdf]

Supporting Information for:

Energy-Screened Many-Body Expansion for Protein–Ligand Interactions: Examining Convergence for Metalloenzymes through Seven-Body Interactions

Paige E. Bowling, Dustin R. Broderick, and John M. Herbert*

March 17, 2026

List of Figures

S1 Upper panels: comparison of MBE(n) accuracy with $M > 0$ versus $M > 1$ as a screening threshold, for (a) 1MMQ and (b) 1ZP5 at the HF-3c level. Lower panels: number of unique subsystems generated for (c) 1MMQ and (d) 1ZP5. Screening thresholds were set to τ_{2B} = 0.25 kcal/mol and τ_{3B} = 0.05 kcal/mol for 1MMQ, and to τ_{2B} = 0.05 kcal/mol and τ_{3B} = 0.05 kcal/mol for 1ZP5. . . . . S2

List of Tables

S1 HF3c Results for 1MMQ as a function of n-body order. S3
S2 HF3c Results for 1ZP5 as a function of n-body order. S4
S3 Aggregate n-body contributions corresponding to the histogram data in Fig. 6 (HF-3c level). S5
S4 ωB97X-V results for 1MMQ in various basis sets. S5
S5 ωB97X-V results for 1ZP5 in various basis sets. S6

*herbert@chemistry.ohio-state.edu

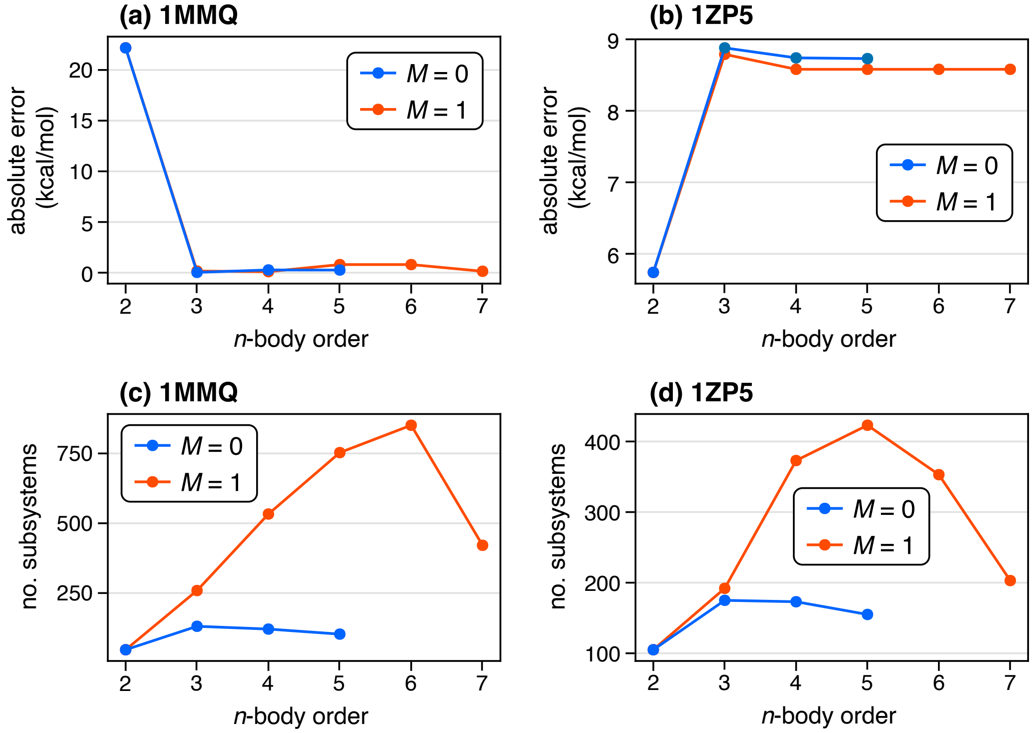

Figure S1: Upper panels: comparison of MBE(n) accuracy with $M > 0$ versus $M > 1$ as a screening threshold, for (a) 1MMQ and (b) 1ZP5 at the HF-3c level. Lower panels: number of unique subsystems generated for (c) 1MMQ and (d) 1ZP5. Screening thresholds were set to $\tau_{2B} = 0.25$ kcal/mol and $\tau_{3B} = 0.05$ kcal/mol for 1MMQ, and to $\tau_{2B} = 0.05$ kcal/mol and $\tau_{3B} = 0.05$ kcal/mol for 1ZP5.

Table S1: HF-3c results for 1MMQ as a function of n-body order.

n	τ_{2B}	τ_{3B}	no distance screening N	|error| (kcal/mol)	|error|/frag. (kcal/mol)	t_{CPU} (h)	$d_2 = 8$ Å N	|error| (kcal/mol)	|error|/frag. (kcal/mol)	t_{CPU} (h)
2	none	none	–	–	–	–	83	21.4	0.13	4.1
3	none	none	–	–	–	–	833	1.0	0.01	69.4
2	0.25	0.025	47	22.2	0.13	2.6	41	22.0	0.13	2.4
3	0.25	0.025	425	0.2	0.00	43.5	187	0.7	0.00	20.8
4	0.25	0.025	1301	0.0	0.00	230.5	491	0.6	0.00	93.8
5	0.25	0.025	2243	0.0	0.00	570.3	733	0.5	0.00	190.8
6	0.25	0.025	2503	0.1	0.00	839.5	687	0.5	0.00	225.9
7	0.25	0.025	1941	0.1	0.00	732.5	241	0.5	0.00	94.3
2	0.25	0.050	47	22.2	0.13	1.9	31	23.7	0.14	1.9
3	0.25	0.050	259	0.2	0.00	27.1	147	0.8	0.00	17.3
4	0.25	0.050	533	0.1	0.00	108.7	343	0.8	0.00	72.2
5	0.25	0.050	753	0.8	0.00	230.3	445	0.7	0.00	133.9
6	0.25	0.050	851	0.8	0.00	333.9	371	0.7	0.00	145.8
7	0.25	0.050	421	0.1	0.00	218.0	159	0.7	0.00	65.3
2	0.50	0.050	31	23.7	0.14	1.9	31	23.7	0.14	1.9
3	0.50	0.050	249	0.9	0.01	27.1	141	0.1	0.00	16.9
4	0.50	0.050	513	0.8	0.00	108.7	331	0.2	0.00	71.1
5	0.50	0.050	753	0.8	0.00	230.3	441	0.1	0.00	133.7
6	0.50	0.050	851	0.8	0.00	333.9	367	0.1	0.00	145.6
7	0.50	0.050	403	0.8	0.00	218.0	153	0.1	0.00	65.0
2	0.75	0.050	27	24.8	0.15	1.8	27	24.8	0.15	1.8
3	0.75	0.050	241	1.3	0.01	26.5	139	0.3	0.00	16.6
4	0.75	0.050	475	1.2	0.01	100.7	413	0.3	0.00	67.6
5	0.75	0.050	661	1.2	0.01	201.4	317	0.2	0.00	124.7
6	0.75	0.050	729	1.2	0.01	283.5	361	0.3	0.00	141.8
7	0.75	0.050	309	1.2	0.01	161.8	147	0.3	0.00	61.3
2	0.75	0.075	27	24.8	0.15	1.8	27	24.8	0.15	1.8
3	0.75	0.075	195	1.3	0.01	22.7	119	0.5	0.00	14.7
4	0.75	0.075	351	1.1	0.01	79.2	251	0.3	0.00	57.9
5	0.75	0.075	485	1.2	0.01	153.9	341	0.4	0.00	108.5
6	0.75	0.075	535	1.2	0.01	204.2	263	0.4	0.00	109.6
7	0.75	0.075	183	1.2	0.01	75.6	117	0.4	0.00	56.8
2	1.00	0.050	27	24.8	0.15	1.8	27	24.8	0.15	0.3
3	1.00	0.050	231	1.1	0.01	25.7	131	0.1	0.00	16.1
4	1.00	0.050	461	1.1	0.01	98.5	309	0.1	0.00	66.8
5	1.00	0.050	653	1.1	0.01	198.8	405	0.2	0.00	122.6
6	1.00	0.050	723	1.1	0.01	281.3	357	0.2	0.00	140.5
7	1.00	0.050	297	1.1	0.01	158.0	137	0.2	0.00	58.8

Table S2: HF-3c results for 1ZP5 as a function of n-body order.

n	τ_{2B}	τ_{3B}	no distance screening				$d_2 = 8$ Å			
			N	|error| (kcal/mol)	|error|/frag. (kcal/mol)	t_{CPU} (h)	N	|error| (kcal/mol)	|error|/frag. (kcal/mol)	t_{CPU} (h)
2	none	none	–	–	–	–	103	5.3	0.03	2.0
3	none	none	–	–	–	–	6059	10.2	0.06	503.0
2	0.05	0.050	105	5.7	0.04	2.2	75	5.5	0.03	1.8
3	0.05	0.050	192	8.8	0.06	12.9	145	8.7	0.05	9.7
4	0.05	0.050	373	8.6	0.05	53.5	263	8.4	0.05	35.0
5	0.05	0.050	423	8.6	0.05	89.5	255	8.4	0.05	52.7
6	0.05	0.050	353	8.6	0.05	77.9	183	8.4	0.05	38.7
7	0.05	0.050	203	8.6	0.05	42.3	131	8.4	0.05	24.5
2	0.10	0.050	85	6.2	0.04	1.9	75	5.5	0.03	1.8
3	0.10	0.050	175	9.2	0.06	12.6	145	8.7	0.05	9.7
4	0.10	0.050	355	9.0	0.06	53.2	263	8.7	0.05	35.0
5	0.10	0.050	405	9.0	0.06	89.2	255	8.4	0.05	52.7
6	0.10	0.050	335	9.0	0.06	77.6	183	8.4	0.05	38.7
7	0.10	0.050	185	9.0	0.06	42.0	131	8.4	0.05	24.5
2	0.25	0.025	47	7.0	0.04	1.3	45	7.3	0.05	1.3
3	0.25	0.025	269	10.0	0.06	18.4	119	9.9	0.06	9.3
4	0.25	0.025	775	10.0	0.06	105.8	233	9.5	0.06	33.8
5	0.25	0.025	1063	9.9	0.06	227.6	229	9.6	0.06	51.6
6	0.25	0.025	1037	9.9	0.06	267.8	155	9.6	0.06	36.9
7	0.25	0.025	813	9.9	0.06	222.6	103	9.6	0.06	22.7
2	0.25	0.050	47	7.0	0.04	1.3	45	7.3	0.05	1.3
3	0.25	0.050	157	10.1	0.06	12.3	119	9.9	0.06	9.3
4	0.25	0.050	333	9.8	0.06	52.2	233	9.5	0.06	33.8
5	0.25	0.050	387	9.8	0.06	88.2	229	9.6	0.06	51.6
6	0.25	0.050	315	9.8	0.06	75.9	155	9.6	0.06	36.9
7	0.25	0.050	165	9.8	0.06	40.3	103	9.6	0.06	22.7
2	0.50	0.050	35	7.5	0.05	1.0	35	7.5	0.05	1.0
3	0.50	0.050	137	10.4	0.07	11.7	103	10.2	0.06	8.7
4	0.50	0.050	275	10.1	0.06	47.0	183	9.9	0.06	28.9
5	0.50	0.050	335	10.1	0.06	78.7	180	9.9	0.06	41.5
6	0.50	0.050	231	10.1	0.06	57.3	77	9.9	0.06	18.2
7	0.50	0.050	129	10.1	0.06	35.3	77	9.9	0.06	18.2
2	0.75	0.050	29	9.2	0.06	1.0	29	9.2	0.06	1.0
3	0.75	0.050	329	11.0	0.07	19.5	101	10.7	0.07	8.5
4	0.75	0.050	265	10.4	0.07	45.2	175	10.3	0.06	27.5
5	0.75	0.050	319	10.4	0.07	74.9	166	10.3	0.06	38.1
6	0.75	0.050	223	10.4	0.07	54.9	73	10.3	0.06	16.3
7	0.75	0.050	121	10.4	0.07	32.8	73	10.3	0.06	16.3

Table S3: Aggregate n-body contributions corresponding to the histogram data in Fig. 6 (HF-3c level).

	1MMQ[a]		1ZP5[b]	
n	N	$\Delta E_{\mathrm{int},n}$[c] (kcal/mol)	N	$\Delta E_{\mathrm{int},n}$[c] (kcal/mol)
2	36	−156.724	61	−70.896
3	102	−21.532	136	3.180
4	164	−0.031	98	−40.040
5	75	0.027	41	−0.057
6	23	0.012	4	0.003

[a]Using $\tau_{2\mathrm{B}}$ = 0.25 kcal/mol, $\tau_{3\mathrm{B}}$ = 0.05 kcal/mol, and $M = 1$. [b]Using $\tau_{2\mathrm{B}}$ = 0.05 kcal/mol, $\tau_{3\mathrm{B}}$ = 0.05 kcal/mol, and $M = 1$. [c]Net n-body contribution to ΔE_{int}.

Table S4: ωB97X-V results for 1MMQ in various basis sets.

		Energy Screening[a]			Energy + Distance Screening[b]		
Basis Set	n	N	ΔE_{int} (kcal/mol)	t_{CPU} (h)	N	ΔE_{int} (kcal/mol)	t_{CPU} (h)
def2-ma-SVP	2	31	−129.5	40	31	−129.5	40
	3	249	−147.7	509	141	−149.0	326
	4	513	−148.0	1876	331	−149.3	1240
	5	753	−148.4	4220	441	−149.5	2497
	6	851	−148.3	6236	367	−149.5	2806
	7	403	−148.3	4124	153	−149.5	1225
def2-ma-TZVP	2	31	−116.2	182	31	−116.2	182
	3	249	−135.8	2250	141	−137.2	1489
	4	513	−135.5	8528	331	−136.9	5715
	5	753	−135.8	18878	441	−137.0	11363
	6	851	−135.7	27142	367	−136.9	12550
	7	403	−135.7	15935	153	−136.9	5279
def2-ma-QZVP	2	31	−115.7	1992	31	−115.7	1992
	3	249	−135.9	24095	141	−137.4	15904

[a]Using $\tau_{2\mathrm{B}}$ = 0.5 kcal/mol and $\tau_{3\mathrm{B}}$ = 0.05 kcal/mol. [b]Using $\tau_{2\mathrm{B}}$ = 0.5 kcal/mol, $\tau_{3\mathrm{B}}$ = 0.05 kcal/mol, and $d_2 = 8$ Å.

Table S5: ωB97X-V results for 1ZP5 in various basis sets.

Basis Set	n	Energy Screening[a] N	Energy Screening[a] ΔE_{int} (kcal/mol)	Energy Screening[a] t_{CPU} (h)	Energy + Distance Screening[b] N	Energy + Distance Screening[b] ΔE_{int} (kcal/mol)	Energy + Distance Screening[b] t_{CPU} (h)
def2-ma-SVP	2	35	−74.5	28	35	−74.5	28
	3	137	−75.1	265	103	−75.0	205
	4	275	−76.5	1093	183	−76.4	691
	5	335	−77.0	1836	180	−76.9	995
	6	231	−77.0	1340	77	−76.8	433
	7	129	−77.0	822	77	−76.8	433
def2-ma-TZVP	2	35	−60.7	135	35	−60.7	135
	3	137	−59.9	1315	103	−59.8	156
	4	275	−61.0	5588	183	−60.8	3541
	5	335	−61.2	9609	180	−61.0	798
	6	231	−61.1	7146	77	−61.0	2342
	7	129	−61.1	4397	77	−61.0	2342
def2-ma-QZVP	2	35	−58.6	1421	35	−58.6	1421
	3	137	−58.1	13611	103	−58.0	10640

[a]Using $\tau_{2\text{B}} = 0.5$ kcal/mol and $\tau_{3\text{B}} = 0.05$ kcal/mol. [b]Using $\tau_{2\text{B}} = 0.5$ kcal/mol, $\tau_{3\text{B}} = 0.05$ kcal/mol, and $d_2 = 8$ Å.
